# Validating an AI-assisted comentoring model for identifying at-risk students and for academic mentoring: a study protocol

**DOI:** 10.3389/fdgth.2026.1738833

**Published:** 2026-03-24

**Authors:** Watson Arulsingh, Praveen Kumar Kandakurti, Mishra Vinaytosh

**Affiliations:** Department of Physiotherapy, College of Health Science, Gulf Medical University, Ajman, United Arab Emirates

**Keywords:** academic mentor, medical education, mentoring, mentorship, risk prediction

## Abstract

**Background:**

Academic mentoring plays a critical role in monitoring student progress, maintaining academic integrity, identifying early signs of risk, and delivering personalized guidance to improve learning outcomes. Traditionally, this has relied on face-to-face interactions; however, advancements in artificial intelligence (AI) have introduced new opportunities for AI-assisted mentoring. While promising, many existing AI models for student monitoring and risk identification are complex and difficult to implement in real-world academic settings. To address this challenge, the present study validates a simplified AI comentor model designed to efficiently identify at-risk students and support continuous academic monitoring focused on pedagogy.

**Methods:**

This study employed a prospective mixed-methods pilot design to evaluate the feasibility, acceptability, and analytic agreement of an AI-assisted assessment framework in medical education. Participants included approximately 40 undergraduate medical students and faculty assessors. Primary outcomes focused on implementation feasibility and acceptability, assessed using structured student and faculty surveys, system-usage metrics, and qualitative feedback. Secondary outcomes evaluated the analytic agreement between AI-derived competency profiles and faculty assessments. The AI component used unsupervised machine learning–based clustering to group students according to multidimensional performance indicators, without prior labels. Agreement was examined using confusion matrices, percentage agreement, and Cohen's Kappa, reported with confidence intervals to account for the exploratory sample size. Given the pilot nature of the study, resampling-based validation (repeated stratified k-fold cross-validation) was used to assess stability rather than definitive diagnostic accuracy. Ethical approval was obtained, and all data were deidentified before analysis.

**Discussion:**

This study will be conducted on a cohort of 40 students from a reputed Health Sciences College, UAE, to evaluate the integrity of a proposed AI comentoring model for monitoring academic performance throughout a semester. The AI models (supervised and segmentation engines) will be tested at two time points: the 5th and 10th weeks. At each time point, categorized student performance data will be uploaded to the AI platform, based on pedagogical parameters, and used to generate a personalized text draft automatically (local pseudonymization + institutional mail merge workflow). To assess the integrity of the used AI, the investigator will perform a manual evaluation of each student's risk status at both checkpoints, alongside statistical analyses. If successful, the system may alleviate the workload of human mentors, enable timely interventions for at-risk students, and enhance overall student performance and retention.

## Introduction

Monitoring the academic progress of students is widely recognized as a valuable form of personalized support, allowing mentees to gain insights from the expertise of their mentors to enhance their learning and achieve their personal goals. However, the growing need to incorporate artificial intelligence (AI) into mentoring within higher education raises critical considerations such as ensuring data integrity, safeguarding system security, maintaining confidentiality, preventing bias, fostering trust, and promoting transparency in algorithms (1).

AI-supported mentoring represents a digitally advanced and scalable approach that replicates the role of mentors by automating tasks such as delivering personalized feedback, providing tailored recommendations, and guiding individual learning paths to improve performance in the academic careers of students (1).

In general, both the face-to-face approach and the AI-supported mentoring approach have their own advantages, disadvantages, and ethical challenges. AI cannot feel or interact with empathy and compassion, which highlights the continued necessity for human teachers and mentors (2). While human mentors can offer personalized support to only a limited number of students, AI-supported mentoring has the potential to broaden access and minimize favoritism.

The unprecedented capabilities of AI risk engines—such as generating human-like text and enabling automated conversations (message generation layer)—carry significant implications across various sectors, including education and healthcare (3), based on each student's unique abilities, interests, and needs, thereby enhancing the learning process (4–13).

The integration of AI into mentorship programs may assist teachers by reducing their workload, allowing them to focus more on creating innovative lesson plans and engaging in professional development. Although data feeding into AI must adhere to key ethical principles, such tasks are vital for real-time monitoring of a large volume of student cohorts, addressing the demands and opportunities of the future (1, 3).

Previous risk prediction models developed by researchers relied on statistical analyses and calculations that were often too complex for faculty to easily understand and apply. Hence, the current study proposes to validate both supervised and unsupervised AI mentoring, using ChatGPT-4 and a predictive risk engine (rules + ML models) to recognize students at risk and provide them mentoring advice to help achieve their learning goals.

The AI Risk Identification Assistance System (AIAS) is conceptualized as a learning analytics early-warning system integrated with mentoring practice. The risk engine identifies students who may not meet the course benchmark (e.g., <65% continuous assessment), while the mentoring layer delivers self-regulated learning (SRL)-oriented prompts and facilitates timely human follow-up at weeks 5 and 10.

The novelty and originality of this project lie in its simplicity and practical application. Earlier studies utilized complex calculations and did not deeply explore how different AI applications align with specific pedagogical models or teaching strategies across disciplines and contexts. They focused on formulas to identify at-risk students and offered automated academic advice through AI. Furthermore, these studies often lacked regular follow-up phases to track student progress and did not test AI-driven mentoring models (14, 15).

Our proposed AI mentoring model is expected not only to recognize at-risk students based on their pedagogical assessment system but also to provide personalized advice to ensure their academic success. This will be achieved through regular automated reminders, risk reports, and academic advice generated by supervised and segmentation engines (unsupervised AI models). The role of the faculty is to simply upload the necessary data to the chatbot. The study will be conducted on students from Health Science at the College of Health Sciences, Gulf Medical University (GMU), UAE, for a semester.

## Materials and methods

### Study design

This study employs a prospective mixed-methods pilot design to evaluate the implementation and exploratory analytic performance of an AI-assisted assessment framework in undergraduate medical education. In line with its pilot intent, the study is designed to separate implementation feasibility and acceptability (primary outcomes) from analytic agreement between AI-derived outputs and faculty assessments (secondary outcomes). The study does not aim to establish diagnostic accuracy or predictive validity but rather to generate preliminary evidence to inform subsequent confirmatory research.

### Study setting and participants

The study was conducted in an undergraduate medical education program at a single academic institution. Approximately 40 medical students enrolled in the target course, and faculty assessors involved in student evaluation were invited to participate. All participants provided informed consent before data collection. Students were assessed as part of routine educational activities. Faculty assessors had prior experience with structured assessment rubrics and received standardized orientation to the study procedures to minimize variability in scoring.

### AI-assisted assessment framework

The AI component consisted of an unsupervised machine learning–based clustering module designed to group students according to multidimensional performance indicators derived from assessment artifacts (e.g., rubric scores, task completion metrics, and structured feedback variables). No outcome labels or performance categories were predefined, and the algorithm did not generate pass/fail or diagnostic classifications. Clustering outputs were interpreted as exploratory competency profiles intended to support sensemaking and reflection, rather than automated judgment. The AI system functioned strictly as a decision-support tool, with all summative decisions retained by faculty.

### Outcome measures

#### Primary outcomes: feasibility and acceptability

Feasibility and acceptability were defined as the primary outcomes of the study. These were evaluated using the following:

- Structured student and faculty questionnaires assessing perceived usefulness, clarity, workload, and trust in the AI-assisted process;
- System usage metrics, including completion rates and interaction frequency; and
- Open-ended qualitative feedback to capture implementation barriers and facilitators.

Survey instruments used Likert-scale items supplemented by free-text responses.

#### Secondary outcomes: analytic agreement

Secondary outcomes focused on exploratory agreement between AI-derived competency clusters and faculty assessment outcomes. Agreement was examined using the following:

- Confusion matrices comparing AI cluster membership with faculty-assigned performance categories;
- Percentage agreement; and
- Cohen's Kappa, reported with 95% confidence intervals.

These analyses were conducted to explore alignment patterns rather than to claim validation or diagnostic performance.

### A sample risk assessment summary by a supervised AI system

A sample CHATGPT-4 automated risk report with a message generation layer [templated or large language model (LLM)-assisted drafting; student-specific academic counseling messages] is provided in the following (Supervised AI mentoring model):

Sample Automated Risk Report by AI (Based on Analysis of Academic Performance)

Dear Student,

Subject: Academic Progress/Student at Risk/Academic Counseling

Based on the latest academic performance evaluation for the course [Course Name – Course Code], you have been identified as at risk of not meeting the passing criteria, as your current performance falls below the required benchmark.

Your academic progress as of [Date] is summarized as follows:

Attendance: 80%

Class Test: 55%

Coursework: 65%

Midsemester Exam: 65%

Total Average Score: 61.67%

[Passing benchmark: You must achieve a minimum score of 65%.]

### Sample-automated unsupervised AI advice

The message generation layer (templated or LLM-assisted drafting) will be addressed to students and copied to mentors as a follow-up to the report.

Subject: Academic Performance Improvement Guidance

Dear Student,

You need to make significant improvements in your coursework and class tests to achieve the minimum benchmark of 65%–70%, as required by your program for successful course completion. Therefore, you must contact your course instructor immediately to seek further assistance and guidance.

The sooner you act on this report, the easier it will be to enhance your grades in the end-of-semester examination, overall course performance, and grade point average (GPA).

### Sample pedagogic AI-generated individualized academic advice/message generation layer (templated or LLM-assisted drafting) for both supervised and unsupervised models

#### Advisory—student performance

The student demonstrates moderate academic performance, with a total average of 61.7%, slightly below the required 65% pass mark. A key area for improvement is the Class Test component (55%), which indicates the need for strengthened conceptual understanding and test-taking strategies.

To improve this component and move past the 65% pass mark, consider the following:

1. Review key concepts regularly—Revisit lecture notes and learning materials to strengthen your understanding of core topics.
2. Practice past papers or sample questions—This helps familiarize yourself with the question style and improve accuracy under time constraints.
3. Clarify doubts early—Seek guidance from your course instructor or peers whenever you find a topic is unclear.
4. Improve time management—Allocate sufficient time for each section during tests to ensure all questions are attempted.
5. Focus on feedback—Analyze errors from previous tests to avoid repeating them in future assessments.
6. Schedule a one-on-one mentoring session—Arrange a one-on-one meeting with your academic advisor for personalized guidance and study plans.

To track individual student progress, similar reports will be generated at three different time points throughout the semester to analyze student improvement. To accomplish this, CHATGPT-4 will be programmed to notify both the mentor and the student if a student’s performance falls below the benchmark at the 5th and 10th weeks of each semester while the data are manually uploaded by the course coordinator for a given course. To support this, ChatGPT will send regular reminders over the following 2 weeks, encouraging students to take necessary actions to enhance their performance and meet the eligibility criteria for the end-semester examinations.

A sample risk identification category was developed to train ChatGPT-4 using structured prompts, enabling the text-generation service to draft mentoring messages from deidentified student performance summaries. An example of the risk profile generated by the unsupervised AI clustering system is presented in Tables 1 and 2. The authors plan to notify students of their cumulative grade point average (CGPA) scores as they transition from one semester to the next (Tables 3, 4) in order to keep the students informed of their academic achievements at each stage. This feature allows students to assess their progress, take proactive steps to improve, or maintain a strong CGPA.

**Table 1:** Sample risk profile identified by an unsupervised AI system.

Area	Performance indicator	Risk level	Comments
Assignment submission	3/5 submitted	High	Two assignments are missing or late
Class attendance	62%	High	The following required 75% attendance
Practical test performance	45%	Medium	Needs improvement in learning demos of hands-on skills
Class test performance	68% average	Medium	Borderline performance
Participation and engagement	Low	High	Rarely engages in class discussions

**Table 2:** Sample performance metrics of a supervised AI risk predictive model.

Assessment component 1 (20% weightage)			Assessment component 2 (20% weightage)			Assessment component 3 (25% weightage)			Analysis and risk identification	
Class test			Coursework			Midsemester Exam			Summative score 65 (excluding End-Semester Exam)	Risk category/performance appraisal
Score above 80% out of 20 (>16)	Score >70%but <80% out of 20 (>14)	Score <60% out of 20 (<12)	Score above 80% out of 20 (>16)	Score >70%but <80% out of 20 (>14)	Score <60% out of 20 (<12)	Score above 80% out of 25 (>20)	Score >70%but <80% out of 25 (>17.5)	Score <60% out of 25 (<15)	Score above 80% out of 65 (>52)	Above average^a^
									Score >70%but <80% out of 65 (>45.5)	Moderate risk: Meet your mentor for appropriate counseling^a^
									Score <60% out of 65 (<39)	High risk. Recommended for rigorous mentor advice, additional remedial measures, and follow-up^a^

^a^ Students will receive an automated checkpoint-based risk classification report at weeks 5 and 10 from chatbot.

**Table 3:** Risk predictive model for the CGPA score.

Program/year/class	Evaluation component		Analysis and risk identification	
	GPA scores secured by students	GPA score conversion to percentage	Risk category	
			90–100	Satisfactory^a^
			85–<90	
			80–<85	Needs to improve in the grade/performance^a^
			75–<80	Moderate risk recommended for mentor advice^a^
			70–<75	
			Less than 70	High risk recommended for rigorous mentor advice, additional remedial measures, and follow-up^a^

^a^ Students will receive an automated checkpoint-based risk classification report at weeks 5 and 10 from chatbot.

**Table 4:** Risk predictive model for assessing attendance and student regularity.

Evaluation component			Analysis and risk identification	
Attendance%			Predicted continuous assessment score 65%	Risk category
			Attendance >85%	Satisfactory^a^
			Attendance >80%<85%	Needs to improve in regularity^a^
			Attendance <80%	High risk suggestive of rigorous mentor counseling and follow-up^a^

^a^ Students will receive an automated checkpoint-based risk classification report at weeks 5 and 10 from chatbot.

### Data processing

This pilot feasibility study focuses on a single group of students from the Bachelor Program at College of Health Sciences (CoHS), GMU, with a minimum sample size of 40 participants to assess the feasibility and acceptability of the approach. Given the pilot sample size (*n*=40), model evaluation will use repeated stratified k-fold cross-validation (or leave-one-out CV) and bootstrap confidence intervals to reduce instability from fixed splits. A pre–post analysis will be conducted to evaluate the significance of differences in the occurrence of high-risk students at each stage of data collection (across three time points). The third assessment point will be their final score and passing percentage. Subsequently, this study may be expanded to a larger sample size using a cluster-randomized group study design. The project will utilize Python and related libraries.

**Figure 1:**
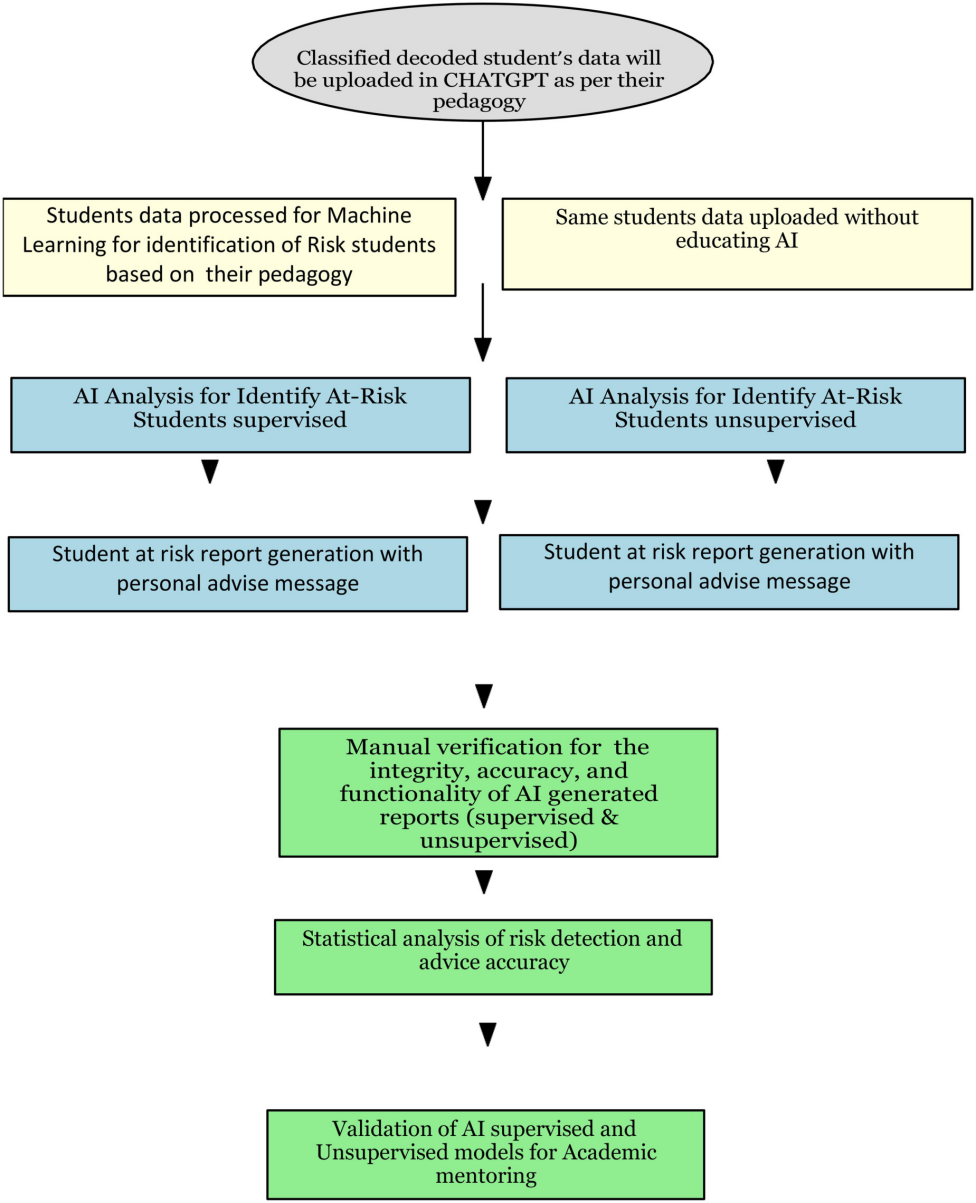
Overview of the intended workflow.

Additional automated alerts and academic advice generated by AI will be thoroughly cross-verified by the investigators with respect to the target students and mentors. In parallel, the investigators will manually analyze student performance using data from the three designated time points [human-in-the-loop governance (mentor approval and escalation)]. This process will allow the investigators to confirm the integrity, accuracy, and functionality of the newly proposed AI mentorship model. A survey will also be conducted among the targeted students and faculty to assess their responses, success rates, and the accuracy of the proposed AI co-mentoring model in tracking academic progress. The present study is designed to use quantitative data, while qualitative data from the survey will be used for validation purposes. Figure 1 provides an overview of the intended workflow in the form of a flowchart.

Student names and contact details remain entirely within existing institutional systems and are never exposed to the AI-assisted system. The system operates on pseudonymized records, using coded identifiers to generate risk labels and draft support messages. Any messages produced are reviewed by staff and, if approved, are delivered through the learning management system or institutional email, ensuring that all student communication occurs within established, secure channels.

Operationally, analysis is conducted on a dataset containing only coded identifiers and learning-related features, while the file linking these codes to student identities is stored separately in an encrypted location. Access to this linkage is restricted to the principal investigator and a designated data steward. The identity key is retained only as long as necessary to support the intervention and is deleted after final grades are released; there is an additional predefined retention period, in line with institutional data governance requirements.

We design the intervention to focus on practical, short-term learning behaviors on which students could realistically act, rather than on generic study advice. Revised messages are grounded in self-regulated learning theory and guide students to set a clear goal for the next 7–10 days, take one or two concrete actions tied to specific course assessments (such as reviewing feedback or practicing a particular skill), and reflect briefly on progress. Each message also provides an explicit invitation to connect with a mentor through office hours or a scheduling link. All along, the tone emphasizes support and next steps, while avoiding deficit-based or stigmatizing language.

To ensure that the approach is feasible and safe in practice, we simplify the workflow and add clear human oversight. Messages are generated from standardized data extracts using a small library of templates, keeping instructor effort manageable. When used, AI support is limited to improving clarity and tone within these templates; all messages are reviewed and approved by a mentor before being sent. We also introduce an escalation pathway so that students showing sustained disengagement or significant performance decline are referred to human support in line with institutional procedures, reducing the risk of harm from false positives or misclassification.

### Data privacy

In this data analysis process using CHATGPT, student identifiers and contact details remain within institutional systems. The AIAS outputs risk labels and message drafts using pseudonymized records. Messages are sent via learning management system (LMS)/institutional email systems after staff review to ensure confidentiality, privacy, and protection from mishandling by fraudulent sources.

### Statistical analysis

Statistical analyses were conducted in alignment with the pilot and exploratory objectives of the study, with clear separation between primary implementation outcomes and secondary analytic agreement outcomes. No analyses were intended to establish diagnostic accuracy or predictive validity.

#### Analysis of primary outcomes

Descriptive statistics were used to summarize feasibility and acceptability outcomes derived from student and faculty surveys and system-usage metrics. Continuous variables were reported as means and standard deviations or medians and interquartile ranges, as appropriate. Categorical variables were summarized using frequencies and percentages. Likert-scale responses were analyzed descriptively and, where relevant, collapsed into agreement categories to facilitate interpretation. Open-ended responses were analyzed using thematic content analysis, with themes reported narratively to contextualize quantitative findings.

#### Analysis of secondary outcomes

Secondary analyses focused on exploratory agreement between AI-derived student clusters and faculty assessments. AI outputs were treated as unsupervised groupings, without prespecified performance labels. Agreement between AI cluster membership and faculty-assigned performance categories was examined using (1) confusion matrices, (2) percentage agreement, and (3) Cohen's Kappa, with 95% confidence intervals

Given the limited sample size, Kappa values were interpreted cautiously, emphasizing direction and magnitude rather than threshold-based validity claims.

#### Resampling and stability assessment

To assess the stability of observed agreement patterns, resampling-based techniques were employed where applicable. Repeated stratified k-fold cross-validation was used to examine the consistency of clustering–assessment alignment across different data partitions. These procedures were not intended to optimize model performance but to evaluate robustness under data perturbation. The results from resampling analyses were summarized descriptively, and variability across folds was reported to reflect uncertainty inherent in small-sample modeling.

#### Sample size and power considerations

Formal power calculations were not performed, as the study was not designed to test hypotheses or estimate diagnostic performance with precision. Instead, the sample size was determined by cohort availability, consistent with pilot-study methodology.

Confidence intervals were reported for agreement statistics to provide transparency regarding sampling variability and interpretability limits. All findings are therefore considered hypothesis-generating and intended to inform the design of larger, adequately powered confirmatory studies.

#### Software and reproducibility

All analyses were conducted using standard statistical and machine learning libraries in Python. Analytical scripts were version-controlled, and parameter settings were fixed before analysis to reduce the researcher's degrees of freedom.

#### Interpretation framework

Analytic results were interpreted within a decision-support paradigm, recognizing faculty assessment as the reference standard and AI outputs as supplementary insights. Statistical findings were used to characterize alignment patterns rather than to adjudicate correctness or automate evaluative decisions.

## Discussion

This study proposes and validates a simplified, integrated AI-assisted comentoring model that combines both supervised and unsupervised machine learning techniques to identify at-risk students and enhance academic performance among Health Sciences students at the CoHS, GMU. By comparing supervised models reliant on labeled datasets and evaluating them using established metrics such as accuracy and precision with a segmentation engine that detects hidden patterns and student groupings, the study aims to determine evaluation feasibility and validity, including agreement with human mentor ratings.

Previous research in academic risk engines utilized algorithms such as k-Nearest Neighbors, decision trees, artificial neural networks, and Naive Bayes classifiers to generate early-stage predictions of academic performance (16). These models often relied on preprocessed datasets and were applied repeatedly throughout the semester to refine predictions and deliver personalized feedback to students. Building upon this, the current model integrates natural language processing capabilities to process large volumes of student data, identify academic risks at an individual level, and generate student-specific messages (17).

The use of AI-powered chatbots such as ChatGPT enhances this model's engagement capacity—particularly in the context of public health and higher education—by offering responsive and context-aware support that adapts to diverse learning needs (18). Furthermore, the comentoring approach automates core mentoring tasks such as tracking attendance, monitoring CGPA progression, and analyzing assignment completion patterns, allowing for more timely and targeted interventions (Table 5). These automated updates and alerts not only inform students of their academic standing but also reduce dependency on remedial coursework by promoting early corrective action.

**Table 5:** Risk mapping.

Mapping variable	Construct	Measure/indicator	Rationale
Assignment submission	Academic self-management	On-time vs delayed submissions	Indicator of organization and time management
Written exam scores	Theoretical knowledge	Percentage score in core physiotherapy modules	Reflects understanding of foundational concepts
Practical skills assessment (OSCE)	Clinical competence	Objective Structured Clinical Examination score	Assesses applied clinical skills
Clinical placement evaluation	Professional competence	Supervisor-rated placement performance	Captures real-world clinical ability
Attendance rate	Engagement	Percentage of sessions attended	Associated with learning outcomes and progression
Grade point average (GPA)	Overall academic performance	Cumulative course grades	Standard indicator of academic achievement

The implementation of this AI comentoring model has several expected benefits. It is anticipated to streamline faculty workload related to student performance monitoring, improve student retention and success through timely support, and foster a proactive mentoring environment. The model's ability to generate real-time, personalized academic advice positions it as a scalable and sustainable solution for improving educational outcomes (19, 20). The Intervention Logic and Escalation Framework is provided in Table 6.

**Table 6:** Intervention logic and escalation framework.

Risk state	Support intensity	Responsible person	Timeline	Escalation criteria
No identified risk	No outreach; routine course communications only	Course team	Ongoing	Not applicable
Early concern (e.g., missed activity, minor performance decline)	Low-intensity supportive message with 1–2 course-specific actions and a short-term goal	Instructor or mentor (message reviewed prior to sending)	Within 3–5 days of trigger	Escalate if there is no response or no improvement within 10 days
Moderate risk (e.g., repeated non-submission and persistent low performance)	Personalized support message plus invitation to meeting or office hours	Instructor or designated mentor	Within 48–72 h	Escalate if the student does not engage or indicators worsen
High risk (e.g., sustained disengagement or marked performance decline across components)	Direct human outreach and referral to institutional support pathways	Program lead, advisor, or student support services	Immediate (≤48 h)	Managed according to institutional support and safeguarding policies

However, the integration of AI tools into educational settings raises valid ethical, technical, and acceptability concerns. While tools like automated graders have been shown to reduce bias and improve assessment fairness (21, 22), their use must be carefully managed to uphold data privacy, transparency, and student trust. This study will address these concerns by adhering to strict data privacy protocols as outlined in the authors' data handling and ethics statement.

Feasibility challenges such as data quality, technical limitations, and the risk of systemic errors will be mitigated through model fine-tuning and human oversight via face-to-face mentoring where needed. Participants will be informed of potential errors issues with AI mentoring, if any, in advance to prevent confusion or panic. A glossary of key terms is appended in Table 7.

**Table 7:** Glossary of key terms.

Term	Definition
AIAS (AI Risk Identification Assistance System)	The AIAS refers to a system in which artificial intelligence tools are used to support and enhance human decision-making or analysis, with final judgment and control remaining with the human user.
Supervised model	A predictive model trained on historical student data with known outcomes, used to classify future student risk.
Unsupervised model	A model that identifies patterns or clusters in student data without prelabeled outcomes, used to detect groups of students with similar risk profiles.
Risk report	A structured summary of a student's likelihood of academic difficulty, including risk labels and recommended support actions.
Human validation	The process by which trained evaluators independently review and confirm risk status, ensuring AI-generated outputs align with expert judgment.

In conclusion, this pilot feasibility study not only explores the comparative strengths of supervised and segmentation engines in risk prediction, but also contributes to the ongoing development of effective validated AI-assisted mentoring systems focused on pedagogy and academic performance outcomes. The study findings are expected to provide critical insights into the practicality, reliability, and impact of integrating AI into student support services and lay the groundwork for future large-scale implementations across educational institutions. Each assessment component has been already mapped to the course learning outcomes.

### Statement on knowledge contribution

This project will create a simplified AI-assisted comentoring model to identify at-risk students early, reduce mentor workload, and provide confidential, individualized guidance. It will also address research gaps by aligning the model with pedagogical principles and including enhanced follow-ups for continuous student support.

### Limitations of the study design

This pilot feasibility study was conducted within a single institution with a single cohort of health science students, and this can be considered a limitation of this study. Future studies will adopt a mixed-methods and qualitatively enriched research design to enhance inclusivity, depth, and contextual understanding of the findings. In addition, subsequent research will involve the systematic use of multiple large language models (LLMs) rather than rely on a single model, enabling comparative evaluation across architectures, training paradigms, and deployment contexts. Leveraging multiple LLMs will improve robustness, reduce model-specific bias, and strengthen the generalizability and reliability of the study's conclusions.

### Dissemination plans

The study results will be disseminated through academic conferences, peer-reviewed journal publications, and institutional reports. Efforts will also be made to share insights with educational policymakers within the institution to inform future educational strategies.

Any amendments to the study protocol will be submitted for review and approval by the institutional ethics committee before implementation.

### Statement for data privacy

Data will be anonymized and the identity will be revealed using a pass key that will be given to the mentor, and a competent faculty with an expertise in using AI will be included in this study. This study will recruit students during Academic Year 2026, with data collection and analysis expected to be completed within a minimum period of 6 months to a maximum of 12 months.

## References

[1] Köbis L Mehner C. Supported mentoring in higher education. Front Artif Intell. (2021) 4:624050. 10.3389/frai.2021.624050 33997774 PMC8120307

[2] Holmes W Bialik M Fadel C. Artificial Intelligence in Education. Boston, MA: The Center for Curriculum Redesign Boston (2019).

[3] Grassini S. Shaping the future of education: exploring the potential and consequences of AI and ChatGPT in educational settings. Educ Sci. (2023) 13:692. 10.3390/educsci13070692

[4] Fazlollahi AM Bakhaidar M Alsayegh A Yilmaz R Winkler-Schwartz A Mirchi N Effect of artificial intelligence tutoring vs expert instruction on learning simulated surgical skills among medical students: a randomized clinical trial. JAMA Netw Open. (2022) 5(2):e2149008. 10.1001/jamanetworkopen.2021.49008 35191972 PMC8864513

[5] Afzal S Dhamecha TL Gagnon P Nayak A Shah A Carlstedt-Duke J AI medical school tutor: modelling and implementation. Proceedings of the Artificial Intelligence in Medicine: 18th International Conference on Artificial Intelligence in Medicine, AIME 2020, Proceedings; Volume 18. Berlin: Springer (2020). p. 133–45.

[6] Chan KS Zary N. Applications and challenges of implementing artificial intelligence in medical education: integrative review. JMIR Med Educ. (2019) 5:13930. 10.2196/13930 PMC6598417 31199295

[7] Francisco RE Oliveira Silva F. Intelligent Tutoring System for Computer Science Education and the Use of Artificial Intelligence: A Literature Review. (2022). Available online at: http://repositorio.grial.eu/handle/grial/2566 (Accessed April 15, 2023).

[8] Klimova B Pikhart M. Exploring the effects of artificial intelligence on student and academic well-being in higher education: a mini-review. Front Psychol. (2025) 16:1498132. 10.3389/fpsyg.2025.1498132 39963679 PMC11830699

[9] Abduljabbar A Gupta N Healy L Kumar Y Li JJ Morreale P. A self-served AI tutor for growth mindset teaching. Proceedings of the 2022 5th International Conference on Information and Computer Technologies (ICICT); 4–6 March 2022; New York, NY, USA. p. 55–9

[10] Kerr P. Adaptive learning. Elt J. (2016) 70:88–93. 10.1093/elt/ccv055

[11] Vieriu AM Petrea G. The impact of artificial intelligence (AI) on students' academic development. Educ Sci. (2025) 15(3):343. 10.3390/educsci15030343

[12] Furini M Gaggi O Mirri S Montangero M Pelle E Poggi F Digital twins and artificial intelligence: as pillars of personalized learning models. Commun ACM. (2022) 65:98–104. 10.1145/3478281

[13] Ambele R Kaijage S Dida M Trojer L Kyando N. A review of the development trend of personalized learning technologies and its applications. Int J Adv Sci Res Eng. (2022) 8:75–91. 10.31695/IJASRE.2022.8.11.9

[14] Garzón J Patiño E Marulanda C. Systematic review of artificial intelligence in education: trends, benefits, and challenges. Multimodal Technol Interact. (2025) 9:84. 10.3390/mti9080084

[15] Lan M Zhou X. A qualitative systematic review on AI empowered self-regulated learning in higher education. NPJ Sci Learn. (2025) 10:21. 10.1038/s41539-025-00319-0 40319057 PMC12049540

[16] Khan I Ahmad AR Jabeur N Mahdi MN. An artificial intelligence approach to monitor student performance and devise preventive measures. Smart Learn Environ. (2021) 8:17. 10.1186/s40561-021-00161-y

[17] Zhou H Shuanghong S Su Y Miao Y Liu Q Zhu L LLM-EPSP: large language model empowered early prediction of student performance. Inf Process Manag. (2026) 63:104351. 10.1016/j.ipm.2025.104351

[18] Abd-Alrazaq AA Alajlani M Alalwan AA Bewick BM Gardner P Househ M. An overview of the features of chatbots in mental health: a scoping review. Int J Med Inform. (2019) 132:103978. 10.1016/j.ijmedinf.2019.103978 31622850

[19] Schlippe T Stierstorfer Q Koppel MT Libbrecht P. Explainability in automatic short answer grading. Artificial Intelligence in Education Technologies: New Development and Innovative Practices: Proceedings of the 2022 3rd International Conference on Artificial Intelligence in Education Technology. Berlin, Germany: Springer (2023). p. 69–87

[20] Ouyang F Wu M Zheng L Zhang L Jiao P. Integration of artificial intelligence performance prediction and learning analytics to improve student learning in online engineering courses. Int J Educ Technol High Educ. (2023) 20:4. 10.1186/s41239-022-00372-4 36683653 PMC9842403

[21] Schlippe T Sawatzki J. Cross-lingual automatic short answer grading. Artificial Intelligence in Education: Emerging Technologies, Models and Applications: Proceedings of the 2021 2nd International Conference on Artificial Intelligence in Education Technology. Berlin, Germany: Springer (2021). p. 117–29

[22] Conijn R Kahr P Snijders C. The effects of explanations in automated essay scoring systems on student trust and motivation. J Learn Anal. (2023) 10:37–53. 10.18608/jla.2023.7801

